# Quantitative Trait Evolution and Environmental Change

**DOI:** 10.1371/journal.pone.0004521

**Published:** 2009-02-20

**Authors:** Mats Björklund, Esa Ranta, Veijo Kaitala, Lars A. Bach, Per Lundberg, Nils Chr. Stenseth

**Affiliations:** 1 Department of Animal Ecology, Evolutionary Biology Centre (EBC), Uppsala University, Uppsala, Sweden; 2 Integrative Ecology Unit, Department of Biological and Environmental Sciences, University of Helsinki, Helsinki, Finland; 3 Department of Theoretical Ecology, Lund University, Lund, Sweden; 4 Centre of Ecological and Evolutionary Synthesis (CEES), Department of Biology, University of Oslo, Oslo, Norway; University of Utah, United States of America

## Abstract

**Background:**

Given the recent changes in climate, there is an urgent need to understand the evolutionary ability of populations to respond to these changes.

**Methodology/Principal Findings:**

We performed individual-based simulations with different shapes of the fitness curve, different heritabilities, different levels of density compensation, and different autocorrelation of environmental noise imposed on an environmental trend to study the ability of a population to adapt to changing conditions. The main finding is that when there is a positive autocorrelation of environmental noise, the outcome of the evolutionary process is much more unpredictable compared to when the noise has no autocorrelation. In addition, we found that strong selection resulted in a higher load, and more extinctions, and that this was most pronounced when heritability was low. The level of density-compensation was important in determining the variance in load when there was strong selection, and when genetic variance was lower when the level of density-compensation was low.

**Conclusions:**

The strong effect of the details of the environmental fluctuations makes predictions concerning the evolutionary future of populations very hard to make. In addition, to be able to make good predictions we need information on heritability, fitness functions and levels of density compensation. The results strongly suggest that patterns of environmental noise must be incorporated in future models of environmental change, such as global warming.

## Introduction

It is being now established that the global environment is changing: the current climate is gradually replaced by a warmer one [1]–[3]. Such large-scale shifts will affect natural systems at several scales and levels of biological organisation. One important aspect is to understand how population will respond to possibly rapid but gradual changes in the environment [4], both on ecological and evolutionary time-scales. It has, for example, been established that the phenology of large-scale bird migration systems has changed in response to climate change, likely as a result of both plastic behavioural and life history responses as well as evolutionary ones [5]. Furthermore, it has been shown that the population of a passerine bird has declined rapidly due to phenological mistiming as a result of climatic change [6]. Although plastic responses within a given trait space of an organism may suffice to accommodate changes in the short-term, adaptive trait evolution is likely and seems necessary if the change is drastic or long-term.

There are many potential factors that might affect a population's adaptation to a moving optimum. From basic evolutionary theory we know that the level of genetic variation is a key factor, as is the width of selection function. A change in the environment does not only influence the phenotypic optimum but also the number of individuals in the population. This in turn affects the degree of intra-specific competition, but also, at the extreme, the ability for individuals to find a suitable mate (Allee effect). Hence, explicit population dynamics considerations must be an integral part of the analysis of evolutionary responses. Furthermore, it is now well-known that environmental fluctuations have different forms depending on the level of serial correlation between years, and that these correlations to a large extent affect population dynamics [7]–[9]. Therefore, all these factors need to be considered when attempting to understand how populations adapt to a changing climate.

There has been theoretical work addressing the question about how a population will adapt to a fluctuating environment [10]–[14]. Here, we will use individual-based simulations of a population with a density-regulated carrying capacity where the environment is allowed to fluctuate in terms of a consistent trend and degree of autocorrelation and where we explicitly analyze the effect of different levels of selection and heritability of a single trait such as body size. We will explore the significance of an environmental trend, such as increasing temperature over time, and assume that there is a phenotypic optimum that changes at each generation. This corresponds to a situation where an environmental variable, for example, temperature, affects fitness in a straightforward way. We will also impose different fluctuations around the trend given by environmental noise of different colours. Thus, over a long time the mean environmental value increases but from one generation to the next the environmental value can change in either direction given by the strength of the temporal autocorrelation of environmental fluctuations. Thus, in this way our models differ substantially from all models published previously. We will change the width of the fitness function to simulate different kind of organisms, from generalists where fitness levels off fairly slowly from the optimal value, to specialists where fitness declines sharply with increasing difference from the optimum. Finally, we will vary heritability as a way to understand how different traits are affected, from life-history traits with a generally low heritability, to morphological ones that tend to have higher heritability.

## Results

**Figure 1:**
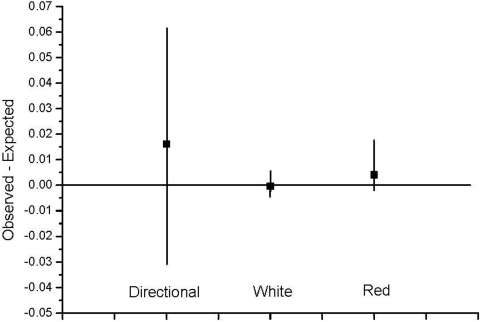
The difference between observed and expected median levels of load for three different models of environmental change. Squares denote median difference and the bars 95% interval of observed values.

We first tested the basic model using the same conditions as in the theoretical models, i.e. directional change (a trend), red noise and white noise, but without the combination of a trend and noise and without density-compensation. When we compared the results from these simulations to the theoretical models, we found that for directional change (trend) the median observed load was slightly higher than expected (Fig. 1). However, a notable feature is that the variance among runs is very large in both directions. The median observed level of load is very close to the expected one with a very small variance for white noise. The same is basically true also for red noise, although the variance is larger with more values on the positive side than on the negative. In short, the simulations produced results largely in agreement with theoretical predictions, using the conditions assumed in the theoretical models.

**Figure 2:**
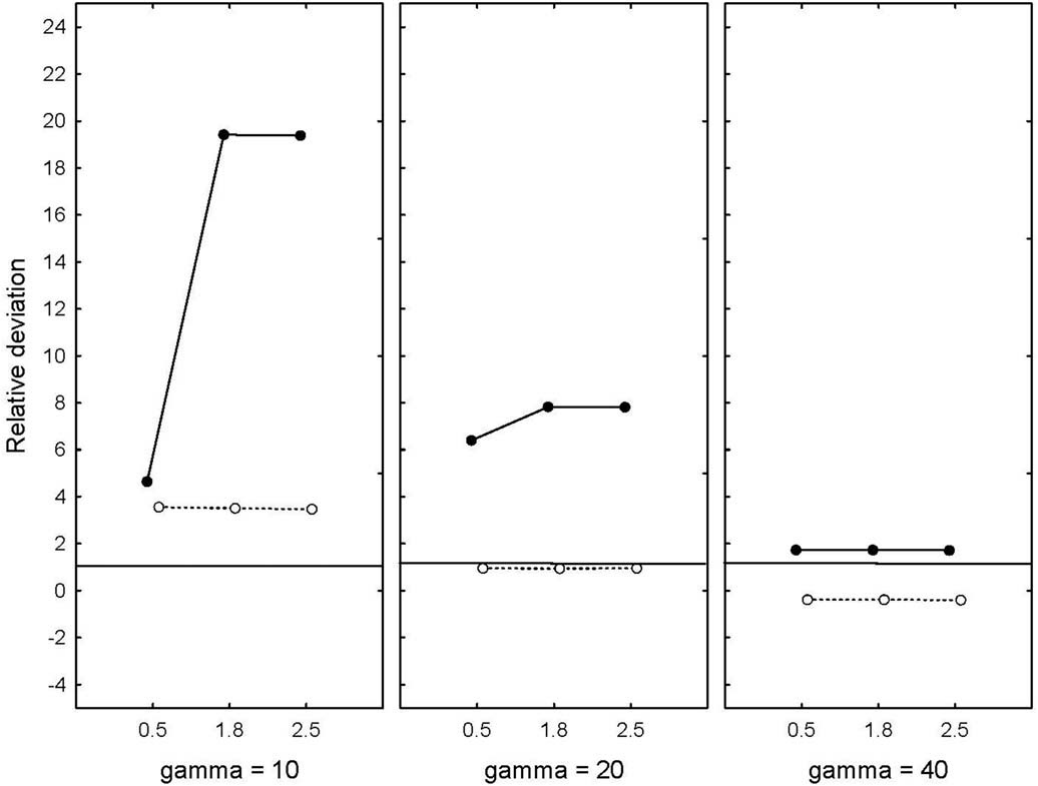
The difference between observed and predicted load expressed as the relative deviation in relation to predicted values for three levels of selection (γ = 10, 20, 40), and levels of density-compensation (α = 0.5, 1.8, 2.2). Solid line is h^2^ = 0.1, and dotted line is h^2^ = 0.5.

In the next step we compared our full model with a trend and different coloured noise added to the trend, and with explicit density-compensation at different rates to the theoretical predictions. The match between observed load and the theoretical predictions varied considerably depending on width of the fitness curve, level of density-compensation and heritability. When the width of the fitness function was wide, predicted and observed values were fairly close, but the observed values were consistently lower than expected when h^2^ = 0.5, and consistently higher than expected when h^2^ = 0.1 (Fig. 2). When selection increased (γ = 20), there was almost no deviation between predicted and observed load when h^2^ = 0.5, but the observed load was about eight times larger than predicted when h^2^ = 0.1. When selection was strongest, the observed load was substantially larger then predicted, and now there is also an interaction with level of density-compensation when h^2^ = 0.1. In particular, load was about 20 times larger than predicted at high levels of density-compensation, but not when r = 0.5. There were no measurable differences between the different kinds of noise (mean relative deviation red noise = 4.9 (SD = 6.41), white noise = 4.3 (SD = 5.53), P>0.5).

**Figure 3:**
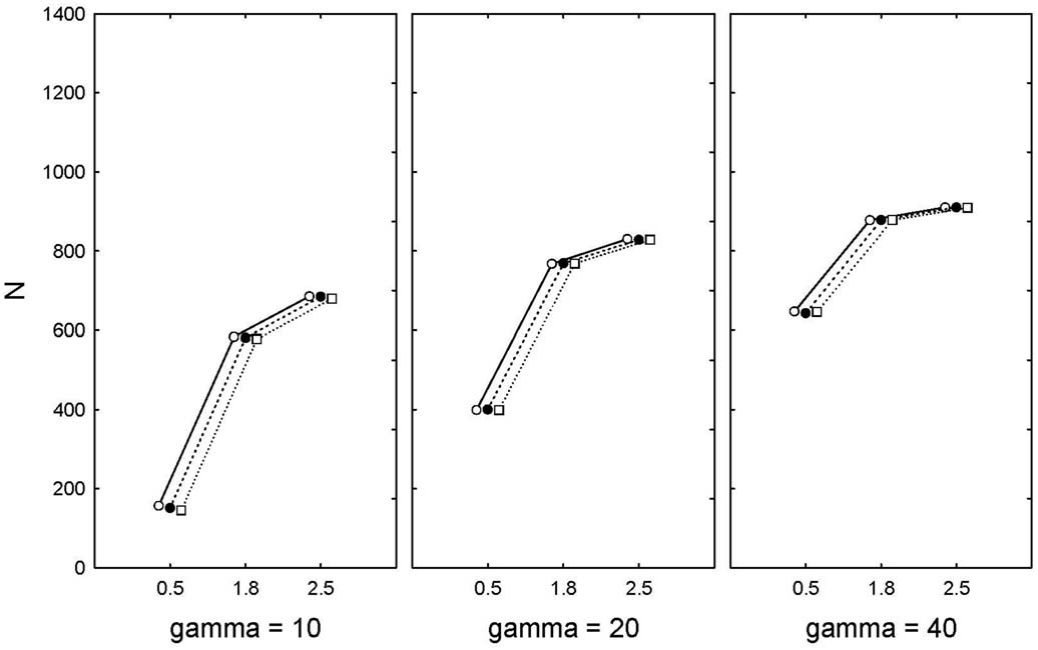
Final population size in relation to levels of selection (γ = 10, 20, 40), and density-compensation (α = 0.5, 1.8, 2.2). The three lines indicate environmental noise (filled circles blue noise, open circles red noise, open square white noise).

Extinctions in our system were found almost only at the lowest amount of density-compensation (α = 0.5), the level strongest selection (γ = 10) and with the lowest heritability (h^2^ = 0.1), where 99.8% of the runs ended in extinction. When α = 0.5, γ = 20 and h^2^ = 0.1, there was a much lower risk of extinction (46.2%). When h^2^ = 0.5 there were almost no extinctions (<2%), and as the fitness function became wider (γ = 40) extinctions were no longer recorded. Final population size was lowest at the lowest levels of density-compensation, and there was a clear interaction between the width of the fitness curve (gamma) and density-compensation (Fig. 3). The effect of environmental noise on final population size was neglible compared to the other factors (Fig. 3).

**Figure 4:**
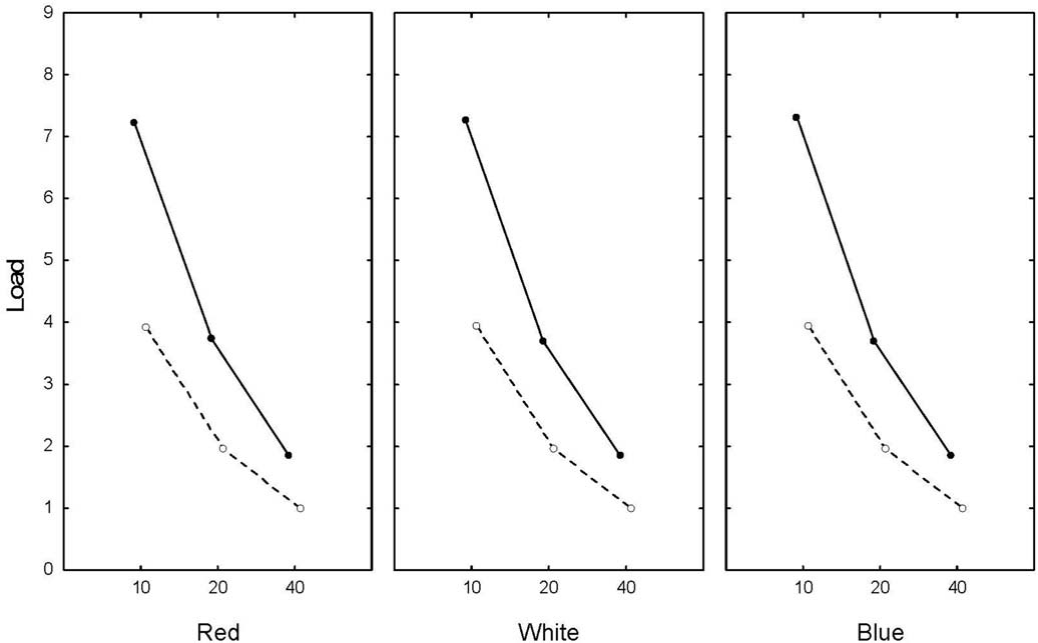
The observed load for the different kinds of environmental noise, and for three levels of selection (γ = 10, 20, 40). Solid line is h^2^ = 0.1, and dotted line is h^2^ = 0.5.

Mean load was affected most strongly by the width of selection function (Fig. 4), but there was also a strong effect of heritability. Thus, load was highest when γ = 10, and h^2^ = 0.1, and lowest when γ = 40 and h^2^ = 0.5. The result was independent on the colour of the environmental noise and the amount of density-compensation (Fig. 4).

**Figure 5:**
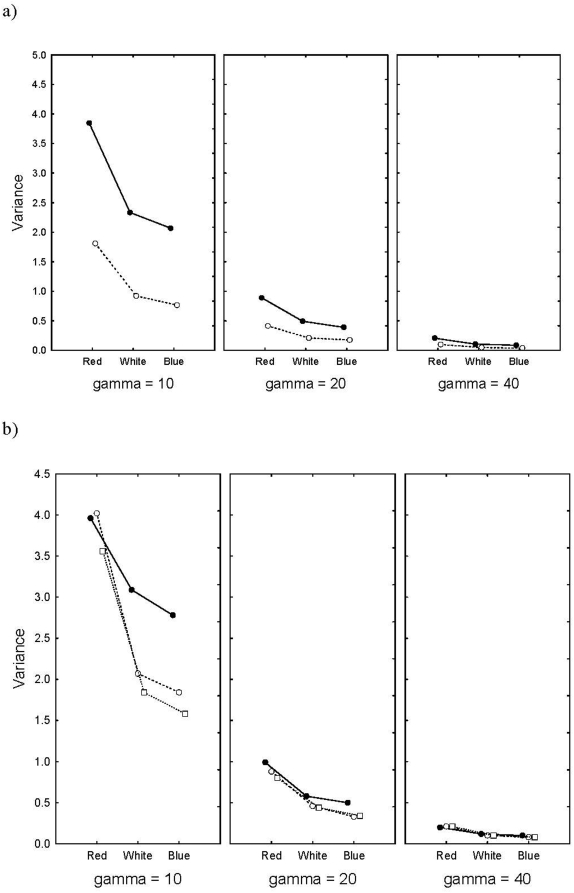
a. The variance in load in relation to level of selection (γ = 10, 20, 40), and kind of environmental noise and different levels of heritability. Solid line is h^2^ = 0.1, and dotted line is h^2^ = 0.5. b. The variance in load in relation to level of selection (γ = 10, 20, 40), and kind of environmental noise and different levels of density-compensation. Solid line is α = 0.5, dashed line α = 1.8, and dotted line α = 2.5.

In contrast, the variance among runs in load was strongly affected by the colour of the environmental noise, and in particular so when the selection was high (Fig. 5a). When γ = 10, the variance for red noise was about twice that for white and blue noise. Again, the effect of heritability was strong. As selection becomes weaker, this effect of noise and heritability vanishes (Fig. 5a, rightmost panel). There was also an effect of density-compensation when selection was strongest (Fig. 5b), where variance in load was independent of level of density-compensation in the red noise runs. Imposing white and blue noise resulted in a higher load when r = 0.5, than at higher levels of density-compensation. No effect of levels of density-compensation or environmental noise could be found when γ = 40.

**Figure 6:**
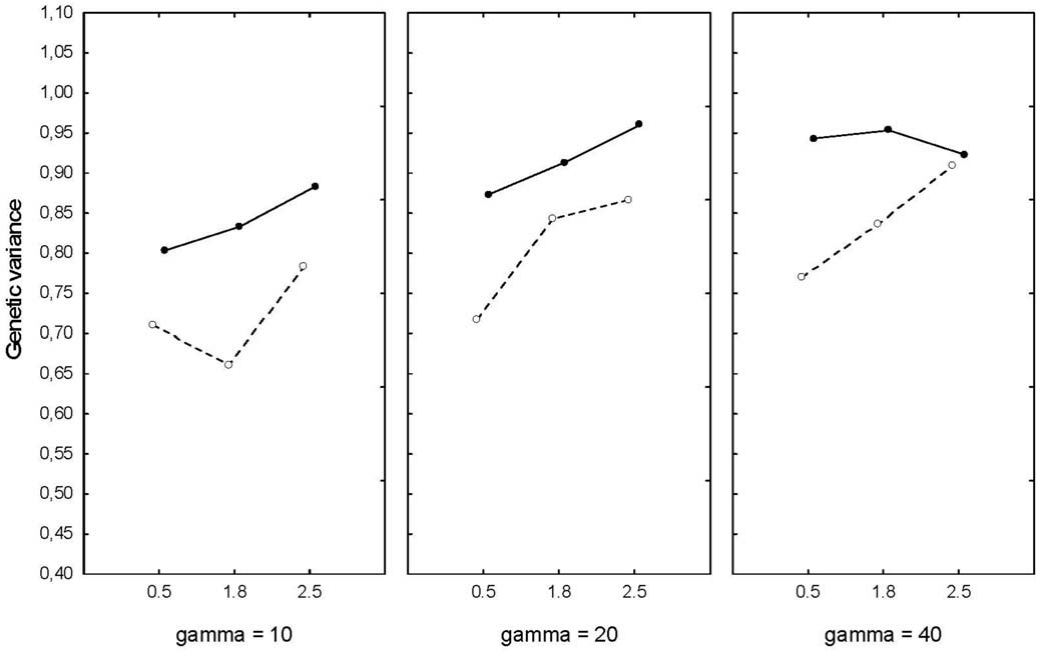
Genetic variance at the end of the runs in relation to levels of selection (γ = 10, 20, 40), and density-compensation. Solid line is h^2^ = 0.1, and dotted line is h^2^ = 0.5.

Genetic variance was reduced in all cases, but this was not affected by the kind of environmental noise. Instead, levels of selection, heritability and level of density-compensation matters (Fig. 6). More variation was lost when heritability was high, in particular when density compensation was low for all levels of selection.

**Figure 7:**
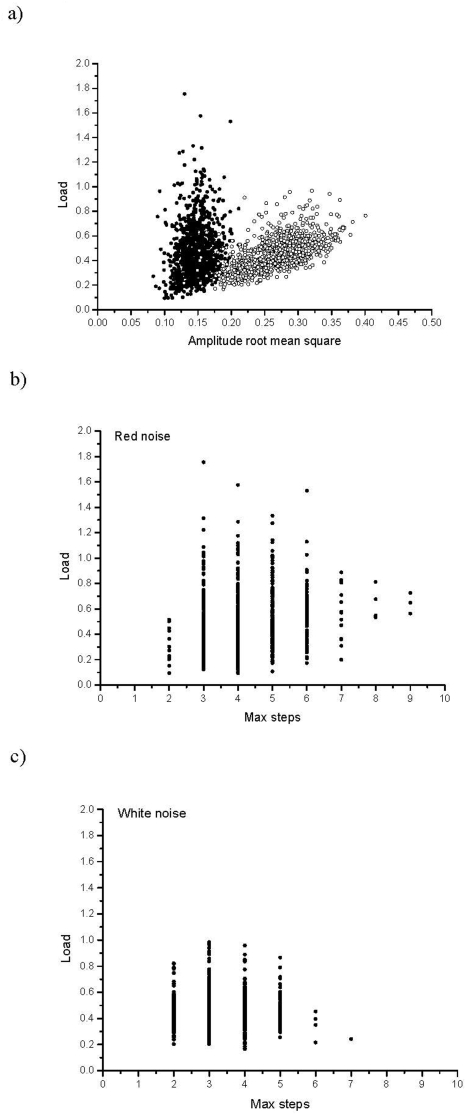
a. Load in relation to amplitude root mean square. Open dots refer to white noise runs, and black dots refer to red noise runs. b. Load in relation to the number of consecutive steps in the same direction for the red noise runs. c. Load in relation to the number of consecutive steps in the same direction for the white noise runs.

To get an understanding of this we looked closer at the difference between red and white noise runs. One possible explanation would be that extreme events have a stronger impact in the red noise case since an occasional abrupt change after a long series of fairly similar conditions makes the population less likely to respond. On the other hand, in the white noise case these events are fairly common. If so, there would be a positive correlation with the probability of extreme events and load for red noise, but not for white noise. The probability of an extreme event scales with the variance in amplitude, or to be more precise, the root mean square of the amplitude (arms, [15]). We found a strong positive correlation between load and arms in the white noise case (r = 0.54, P<0.001, N = 1000 runs), and a weaker correlation in red noise case (r = 0.18, P<0.001, N = 1000 runs, Fig. 7a). Even if there is a correlation with red noise the pattern found does not match the predictions well. On the other hand, if we look at the largest number of generations changing in the same direction, there is a positive correlation with load for red noise (r = 0.14, P<0.001, N = 1000, Fig. 7b) but not for white noise (r = −0.02, P = 0.49, N = 1000, Fig. 7c).

## Discussion

The most important factors determining load were width of selection function and the level of heritability, whereas environmental noise and levels of density-dependence had only a small impact. However, when we consider the variance among runs in load, then environmental noise becomes important, but there is a strong interaction with selection levels and this is most prevalent when the fitness function is narrow (strong selection). The level of density-dependence matters as well, but only when selection is strong. The level of density-dependence is most important for population size, but apparently much less so for magnitude of load, and only partly to the variance in load.

What is new for this study compared to the theoretical studies is the combined effect of a trend, environmental noise and different levels of density-compensation. In particular, neither final population size nor load was affected by the pattern of environmental fluctuations, but variance among runs in load was. When we imposed red noise variance was almost twice as high as if we imposed blue or white noise. This was very clear when the width of selection function was narrow, but disappeared with when selection becomes weaker. There was also an interaction between level of density-dependence and the colour of noise when selection was strong. In the red noise scenario there was no difference dependent on level on density-dependence, but there was a strong effect in the white and blue noise simulations. With a higher variance when density-dependence was low compared to the two higher levels. Again, this effect disappeared with decreasing width of selection function. It is also clear that the theoretical expectations derived by [13] were accurate only under some conditions given by the assumptions of these models, but clearly inaccurate under other conditions; in particular when the fitness function was narrow (Fig. 2). In general, when we added red noise the result was much less predictable than if we added white or blue noise. Lande and Shannon [13] argued that the combined effect of a trend and noise was additive, but this was clearly not the case in our simulations.

Imposing a trend on a population results in a load and the magnitude of load is determined by the strength of the trend. If red noise is imposed on the trend we add another level of trends which further increase load. Since the long trends are not always found we get a variance in the overlaying trends that results in an increased variance in load. This is not the case with white noise, where overlaying trends are shorter and the variance is smaller, and thus the variance among runs is lower. Thus, red noise results in more unique sets of conditions than white noise, and this is visible in the variance among runs in load.

The presence of red noise has been shown to have strong effects on, for example, risk of extinction for the very same reason [7]–[9], [16], [17]. This means that the exact pattern of environmental change matters for how well we can predict the evolutionary response to a changing environment. Many environments are characterised by red noise [18], and for example, in Europe many species are affected by the North Atlantic Oscillation (NAO) pattern, which is positively autocorrelated [16]. One factor that is unexplored in this context is the interaction between large-scale (‘global’) fluctuations, such as NAO, and local fluctuations due to small-scale changes in climate or biotic interactions.

The level of heritability was found to be very important, and with a low heritability (h^2^ = 0.1) load was higher than with a higher heritability (h^2^ = 0.5). Heritability is a measure of the correlation between genotype and phenotype, and since selection act on phenotypes, this correlation matters for the evolutionary response, which is clear from [11]. If the correlation is weak, then the phenotypes selected are not necessarily the optimal genotypes. In biological terms, this means that traits with low heritability, such as most life-history traits, will respond slowly and may lag behind the optimum quite considerably, while traits with a higher heritability, such as most morphological traits, respond faster and are less displaced from the optimum in this scenario. In contrast, genetic variance is lost slower when heritability is low. Again, this is in accordance with main theory; if the correlation between phenotype and genotype is low, and since selection acts on phenotypes, the effects of selection becomes weak at the genotypic level.

The width of the fitness function (“strength of selection”) affected the results considerably. In general, when the fitness function was narrow load was higher, but the pattern is far from straightforward as this also depends on the level of heritability. Furthermore, when it comes to the variance among runs, environmental noise and the level of density-compensation matters, in the latter case only when heritability is low. These results are expected since the width of the fitness curve determines the penalty in terms of fitness for being less-than-optimal. In biological terms this means that species with a narrow fitness function, i.e. specialists, will have it harder to cope with changes than generalists, i.e. species where the fitness function is broader. Consequently we found extinctions being almost entirely confined to cases where the fitness function was narrow, but none when the function was wide. The effect of selection on population size is important. When the optimum moves mean fitness is reduced and hence population size decreases. This can to some extent be mediated by density-compensation for example reduced competition for food, but not entirely. In a species where the optimum is strictly affecting the physiology of the organism, such as for temperature-dependent life-history traits in many insects [19], the presence of other individuals does not matter much compared to the environmental cues. This means that an optimum that is moving would result in decreased population sizes and an increased risk of extinction. In fact, we did preliminary runs with a higher per generation change in optimum (e = 0.15), which resulted almost exclusively in extinctions, in particular at higher levels of selection, but also in the other cases.

This importance of stochastic effects was stressed by [12] who argued that environmental changes above 10% of the phenotypic standard deviation per generation would certainly lead extinction, and perhaps even as little a 1% might be enough. In our study we used 5%, and found that when heritability was low, density-compensation was low, and with a narrow fitness function, the probability of extinction was almost 100%. However, increasing heritability to 0.5, made caused the probability of extinction to drop to zero, as did most other changes. Preliminary simulations (not shown) with larger changes than 10% per generation almost invariably led to extinctions in accordance with the result of [12].

The results point to several disturbing factors for the understanding of the evolutionary effects of a climatic change. For example, we need to know something about the level of density-compensation in a certain population, i.e., a set of basic ecological data lacking for most species and a phenomenon that is notoriously difficult to estimate using field data. For example, to be able to measure environmental stochasticity based on population size data we need at least 15 years of data at the individual level to get good enough parameter estimates [20]. Likewise, we need to estimate which traits are the ones affecting fitness mostly, including their heritabilities. This model is a simplification even though the results are complex, and factors such as genetic correlations between traits, and genotype-environment interaction can potentially affect the rate of adaptation to novel climatic conditions. We have chosen to use one trait such as body size, which is known to affect fitness in many species (e.g. [19]), and which is a composite trait. Genotype-environment interaction can drastically affect the levels of variation for selection to act on, and on the evolutionary response [21]. Since the level of heritability was shown be very important in determining the magnitude of load, factors such as plasticity and genotype-environment interacts that can affect heritability are clearly important.

However, we have chosen in this analysis not to include this aspect in order to show that even without this complicating factor the outcome of a directional change in environmental conditions is very hard to predict without knowledge of a number of basic ecological and genetic parameters. Genotype-environment interactions are certainly important, but also notoriously difficult to model since the interaction can take any form, and indeed evolve itself. One obvious way to get an understanding of this is to measure the variance in the shape of the reaction norms, in addition to the shape itself [22], [23], but the empirical data is lacking here.

Even if all this is measurable in any natural population, we also need to know the colour of the environmental noise, and most likely also the exact sequence of event for an accurate prediction. If the environmental noise has a positive autocorrelation our results show that the details of the sequence of environmental change have a strong impact on the ability of the population to adapt to the changing conditions. This is a new result for this study and generally overlooked in this kind of studies. Even though models incorporating different noise have been developed [13], the combination of a trend with different kind of noise has not been explored before to our knowledge. The results convincingly show that this is necessary. This also means that in a real world forecast climatic models needs to be developed and incorporated in detail into the biological considerations. Understanding the change in mean environmental cue (temperature, precipitation etc.) is obviously important, but so is also the pattern of the variance around the mean.

## Materials and Methods

We used individual based simulations of a finite and homogenous population with random mating among sexually reproducing individuals. Each run was initialized with 1000 individuals assigned genotypes (*a*_i_) randomly drawn from a normal distribution with zero mean and unit standard deviation *N*(0, 1). Phenotype *i* (*z*_i_) was created by adding a random number (*e_i_*) from a *N*(0, *P*) distribution:
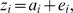
(1)where *P* was scaled to give different levels of heritability, *h^2^* = var(*a*)/var(*z*). We assume that the allelic effects are additive and Gaussian. The variance of genotypic values is therefore the additive genetic variance, V_A_.

Fitness (*w*) for individual *i* was determined as:
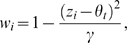
(2)where θ_*t*_ is the environmentally optimal phenotype at time *t*, and *γ* is a factor determining the width of selection function (width of the fitness curve), where selection increases with decreasing *γ*. A similar fitness function is used, e.g., by [12], and this form gives numerically the same results. Scaling in this way makes maximum fitness = 1. This means that an optimally adapted individual replaces itself when the population size is stable.

Individuals were then randomly assigned a sex and mated randomly. The fitness of a pair is the sum of the fitness values of the two parents (w_sum_). To incorporate density-compensation, fitness of each individual was scaled as follows
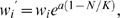
(3)where *α* is a parameter describing the strength of density-dependence (larger values of *α* mean stronger effect of density dependence), *N* is the population density and *K* is carrying capacity. The higher *α*, the stronger is the effect of increased population density *N*. We used three levels of *α*: 0.5, which is weak density compensation, 1.8 that gives strong density-compensation but no cyclic or chaotic dynamics, and 2.5 that is strong density-compensation. Since the dynamics of populations is fundamentally different at these three levels of density-compensation any difference in outcome due to density-compensation would be apparent using these levels.

To incorporate demographic stochasticity we assigned the number of offspring according to a Poisson(*w_sum_*) distribution. Offspring genotypes had an expectation equal to the mean parent genotype with a variance equal to half the genetic variance of the parents (1/2 * Var[a_m_, a_f_]) [24]. We then created phenotypes by adding an environmental component in the same way as when the initial population was created keeping the level of heritability constant. This is justified on the basis of the models of [25], who demonstrated that a most likely cause of the environmental variation is determined by the cost of minimising variability during development. This has the effect that as the variance among genotypes decrease due to selection, so does the variation in terms of different ability to minimize developmental errors, and hence the heritability will stay constant, or at least nearly so. All adults died and the new generation was entirely set by the offspring generated.

At the start of the simulation the population had a mean value equal to the environmental optimum (locally adapted). The initial quality of the environment was assigned to be Q_0_ = 0. We then added an environmental trend by each generation adding a small number (0.04) to the environmentally optimal value of the phenotype. This number is arbitrary as we are only interested in the general differences between different environmental scenarios when there is a trend in the changes. We also added stochastic noise around the mean value of the environmental optimum; white noise from a *N*(0,1) distribution, red noise with an autocorrelation of 0.7, with the range −1 to 1, and blue noise with an autocorrelation of −0.7, with the range −1 to 1 [17].

The simulation was run for 30 generations and at each generation we recorded the difference between the phenotypic mean value of the population and the environmental optimum and calculated the evolutionary *load* [13] defined as
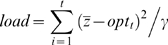
(4)which is the difference between the population mean at a given generation and the optimum summed over all generations (*t*). We also calculated the variance of load in each case. In addition, we also recorded the difference between the population mean and the optimum at the end of the time period simulated.

Each parameter combination was replicated 2000 times. In addition to the three levels of *α*, we used three different values of *γ*: 10, 20, and 40, where 10 is strong selection and 40 is very weak selection. To visualise these numbers, when *γ* = 10 individuals being 1 SD larger or smaller than the mean have a fitness only 60% of maximum, *γ* = 20 means 90% of maximum, and *γ* = 40 means 95% of maximum. These values correspond qualitatively to the values in [26], where 20 represent the median selection intensity measured in natural populations, 10 is found in less than 5% of empirical data sets, and 40 is used as lower level commonly found in nature. We used two values of heritability: 0.1 and 0.5. The first value corresponds to many life-history traits, and the second value to most morphological traits [27]. Initial population size and *K* was kept at 1000 in every run.

The expected load under various patterns of environmental change has been derived theoretically [11], [13]. For sustained directional change (trend) the expected load is 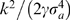, where *k* is the rate of directional change (0.04 in this paper), and 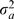 is the additive genetic variance. For a fluctuating environment with white noise the expected load is 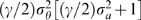, and for red noise the expected load is 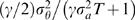, where *T* is the autocorrelation time [13]. There are no expectations derived for a trend with environmental noise (red or white), but [13] argued (without proof) that the different expectations should be additive.

We will first compare the results from our model with the theoretical expectations using the same models of environmental change. This will work as a test of the model itself, but also add information on the variance of the expectations when *N* is finite, and demographic stochasticity is added. Next, we will compare the observed levels of load for the different scenarios of environmental trends (see above) to that expected. We calculated the proportion of runs that were above or below expected, and if this was lower than 0.05 the result was treated as significant. We will then show the actual levels of load, the variance, and mean difference at the end of the simulations.
